# Updated evidence-based bowel management among spinal cord injury patients

**Published:** 2012-11

**Authors:** Radin Maheronnaghsh, Ali Yousefian, Vafa Rahimi-Movaghar

**Affiliations:** ^a^Sina Trauma and Surgery Research Center, Tehran University of Medical Sciences, Tehran, Iran.; ^*b*^Research Centre for Neural Repair, University of Tehran, Tehran, Iran.

## Abstract

**Background:**

Bowel and gastrointestinal dysfunctions are common post- spinal cord injury (SCI) complications affecting both physical and psychological aspects of the quality of life. More common and important gastrointestinal problems include constipation and fecal retention, constipation with frequent incontinence, hemorrhoids and serious abdominal complications such as cholecystitis, upper gastrointestinal bleeding, pancreatitis, appendicitis and the superior mesenteric artery syndrome. Therefore, the present study aims to review current evidence-based methods for bowel management among SCI patients.

**Methods:**

The MedLine/PubMed was used as the data source to identify studies published from January 1, 1995 to August 1, 2012. Cross referencing of discovered articles was also performed. All of the evidences related to bowel management in SCI patients were extracted. We designed search strategies using following keywords in both text word and subject heading forms: Constipation, incontinence, fecal retention, hemorrhoids, superior mesenteric artery syndrome, spinal cord injury, veteran, evidence, assessment, prevention and treatment. According to the inclusion criteria, 122 studies were selected for further analyses.

**Results:**

For Constipation and fecal retention, a consistent and structured regimen was recommended. For constipation with frequent incontinence several methods were investigated such as digital rectal stimulation, abdominal massage, deep breathing , Valsalva maneuver, forward-leaning position, oral bowel medications, regular diet, Prokinetic medications, Prucalopride, Fampridine, colostomy, sacral electrical stimulation, functional magnetic stimulation, neuromuscular electrical stimulation and posterior tibial nerve stimulation. Hemorrhoids can be prevented THROUGH suppositories, enemas, digital rectal stimulation, stool softeners, minimizing trauma, and topical anti-inflammatory creams. The superior mesenteric artery syndrome would be treated by conservative management.

**Conclusions:**

In addition to prevention, training plays an important role in bowel management among SCI patients. Although different prevention methods have been reported, there are few well designed studies for managing gastrointestinal (GI) complications. In conclusion, training plays an important role in preventing bowel complications among SCI patients. However, more investigation is necessary to develop more evidence-based techniques to approach and manage GI complications in individuals with SCI.

**Keywords:**

Constipation, Fecal incontinence, Hemorrhoids, Superior mesenteric artery syndrome, Spinal cord injury


					Volume 4, Suppl. 1 Nov 2012
Publisher: Kermanshah University of Medical Sciences
URL: http://www.jivresearch.org
Copyright:
Congress of Iranian Neurosurgeons, 14-16 November 2012, Kermanshah, Iran
Paper No. 59
Updated evidence-based bowel management among spinal
cord injury patients
Radin Maheronnaghsh a
, Ali Yousefian a
, Vafa Rahimi-Movaghar a,b,*
a
Sina Trauma and Surgery Research Center, Tehran University of Medical Sciences, Tehran, Iran.
b
Research Centre for Neural Repair, University of Tehran, Tehran, Iran.
Abstract:
Background: Bowel and gastrointestinal dysfunctions are common post- spinal cord injury (SCI) complications
affecting both physical and psychological aspects of the quality of life. More common and important gastrointestinal
problems include constipation and fecal retention, constipation with frequent incontinence, hemorrhoids and serious
abdominal complications such as cholecystitis, upper gastrointestinal bleeding, pancreatitis, appendicitis and the
superior mesenteric artery syndrome. Therefore, the present study aims to review current evidence-based methods
for bowel management among SCI patients.
Methods: The MedLine/PubMed was used as the data source to identify studies published from January 1, 1995 to
August 1, 2012. Cross referencing of discovered articles was also performed. All of the evidences related to bowel
management in SCI patients were extracted. We designed search strategies using following keywords in both text
word and subject heading forms: Constipation, incontinence, fecal retention, hemorrhoids, superior mesenteric artery
syndrome, spinal cord injury, veteran, evidence, assessment, prevention and treatment. According to the inclusion
criteria, 122 studies were selected for further analyses.
Results: For Constipation and fecal retention, a consistent and structured regimen was recommended. For constipation
with frequent incontinence several methods were investigated such as digital rectal stimulation, abdominal massage,
deep breathing , Valsalva maneuver, forward-leaning position, oral bowel medications, regular diet, Prokinetic
medications, Prucalopride, Fampridine, colostomy, sacral electrical stimulation, functional magnetic stimulation,
neuromuscular electrical stimulation and posterior tibial nerve stimulation. Hemorrhoids can be prevented THROUGH
suppositories, enemas, digital rectal stimulation, stool softeners, minimizing trauma, and topical anti-inflammatory
creams. The superior mesenteric artery syndrome would be treated by conservative management.
Conclusions: In addition to prevention, training plays an important role in bowel management among SCI patients.
Although different prevention methods have been reported, there are few well designed studies for managing
gastrointestinal (GI) complications. In conclusion, training plays an important role in preventing bowel complications
among SCI patients. However, more investigation is necessary to develop more evidence-based techniques to
approach and manage GI complications in individuals with SCI.
Keywords:
Constipation, Fecal incontinence, Hemorrhoids, Superior mesenteric artery syndrome, Spinal
cord injury
* Corresponding Author at:
Vafa Rahimi-Movaghar: Associate professor of Neurosurgery, Research Deputy, Sina Trauma and Surgery Research Center, Sina Hospital, Hassan-
Abad Square, Imam Khomeini Ave, Tehran University of Medical Sciences, Tehran, Iran. Phone: (+98) 915 342 2682, (+98) 216 6757010,
Fax: (+98) 216 675 7009, Email: v_rahimi@sina.tums.ac.ir , v_rahimi@yahoo.com, (Rahimi-Movaghar V.).

				

